# Subcortical Effects on Voice and Fluency in Dysarthria: Observations from Subthalamic Nucleus Stimulation

**DOI:** 10.4172/2161-0460.1000392

**Published:** 2017-10-30

**Authors:** Diana Sidtis, John J Sidtis

**Affiliations:** 1 Department of Communicative Disorders, New York University, New York 10012, USA; 2 Brain and Behaviour Laboratory, Geriatrics Division, Nathan Kline Institute for Psychiatric Research, Orangeburg, New York 10962, USA; 3 Department of Psychiatry, NYU Langone Medical Center, New York 10016, USA

**Keywords:** Parkinsonian dysarthria, Speech intelligibility, Deep brain stimulation, Speech task, Acoustic measures, Basal ganglia, External vs. internal models for motor behaviors

## Abstract

**Objective:**

Parkinson’s disease (PD), caused by basal ganglia dysfunction, is associated with motor disturbances including dysarthria. Stimulation of the subthalamic nucleus, a preferred treatment targeting basal ganglia function, improves features of the motor disorder, but has uncertain effects on speech.

We studied speech during contrasting stimulation states to reveal subcortical effects on voice and articulation. Measures were made on selected samples of spontaneous and repeated speech.

**Methods:**

Persons with Parkinson’s disease (PWP) who had undergone bilateral deep brain stimulation of the subthalamic nucleus (DBS-STN) provided spontaneous speech samples and then repeated portions of their monologue both on and off stimulation. Excerpts were presented in a listening protocol probing intelligibility. Also analysed were a continuous phrase repetition task and a second spontaneous speech sample. Fundamental frequency (F0), harmonic-to-noise ratio (HNR), jitter, shimmer and fluency were measured in these three speech samples performed with DBS stimulation on and off.

**Results:**

During subcortical stimulation, spontaneous excerpts were less intelligible than repeated excerpts. F0 and HNR were higher and shimmer was decreased in repetition and stimulation. Articulatory dysfluencies were increased for spontaneous speech and during stimulation in all three speech samples.

**Conclusion:**

Deep brain stimulation disrupts fluency and improves voice in spontaneous speech, reflecting an inverse influence of subcortical systems on articulatory posturing and laryngeal mechanisms. Better voice and less dysfluency in repetition may occur because an external model reduces the speech planning burden, as seen for gait and arm reach. These orthogonal results for fluency versus phonatory competence may account for ambivalent reports from dysarthric speakers and reveal the complexity of subcortical control of motor speech.

## Introduction

Since deep brain stimulation of the subthalamic nucleus, first introduced as a medical treatment for Parkinson’s disease (PD) in 1987, was approved in 1997 by the FDA, it has become an established therapy of choice for tens of thousands of individuals world-wide with PD and other neurological or psychiatric disorders [1,2]. PD is caused by basal ganglia disease and leads to motor disturbance, including dysarthria, and deep brain stimulation has a dramatic effect on subcortical function. This scenario presents a natural experimental setting for studying subcortical effects on two important components of speech, voice and fluency. The present study examines the effects on speech of stimulatory intervention in subcortical disease. Both spontaneous and repeated speech, obtained under controlled conditions with and without stimulation states, were analysed using listening studies, acoustic measures, and fluency analyses. The focus is on the effects of subcortical stimulation therapy on voice and articulation [3].

The physiological basis for the positive effects of deep brain stimulation on motor systems is not well understood [4-8] but there is a general consensus in the PD community that the therapy reliably reduces tremor and bradykinesia and provides for more consistent symptom control [9-11]. The therapeutic effectiveness of this treatment may allow a dose reduction of PD medications or lessen the need for increased doses. The medications, with prolonged use, are often associated with dyskinesias [12-14], adventitious movements of face and arms that can interfere with activities of daily living.

Dissociation between corporeal motor behaviours and speech in subcortical disease has been reported. Sustained phonation and monologue speech were impaired during high frequency stimulation, while motor scores improved [15]. From some observations, it appears that laryngeal and articulatory functioning is outside of dopaminergic control in persons with Parkinson’s disease (PWP) [16]. This view is supported by a longitudinal study of prosodic impairment, in which the speech measures did not correlate with motor scores in the PWP study group [17,18], likely due to differences in the role of dopaminergic processes in the regulation of speech and limb movements [19,20]. Similarly, the improvement in motor difficulties afforded by deep brain stimulation does not predictably extend to speech.

## Effects of Subcortical Stimulation in Speech: An Overview

The effects of stimulation therapy, as seen in quantitative and qualitative measures of speech and intelligibility, have often yielded post-surgical speech intelligibility deterioration [21-27]. Inconsistencies in published and patient reports, as well as a variety of approaches to the question, are prevalent [28]. In a meta-analysis of studies of speech following deep brain stimulation surgery using a variety of speech tasks and motor speech measures, four of seven studies revealed negative effects, but individual differences were noted [29]. No change or improvement in speech is also seen following stimulation therapy [30-32].

When improvements in association with stimulation are noted, they often reference or include voice quality. Laryngeal vocal fold mobility was affected by DBS in a single PWP [33]. A voice disorder forms part of the PD speech disability profile; variable fundamental frequency, hypophonia, monotone, and breathiness. Examining samples of steady phonation and repetition in one individual, stimulation resulted in improved measures of F0, jitter and shimmer, harmonics-to-noise ratio, speech rate, intensity and duration [34]. Wang et al. [35] observed increased intensity, a benefit to the hypophonic Parkinsonian speaker, in sustained vowel production. An increase in harmonic-to-noise ratio (HNR) in periodic portions of speech was said to reflect more efficient function of vocal fold vibration and articulatory setting with subcortical stimulation [20,36,37]. Reduced vocal tremor [38], increased maximum phonation time [32,39,40], better stability of pitch and or amplitude [32,39], and increases in F0 variability and intensity [34,41-43] have also been associated with stimulation therapy. Some studies reported improvements for prosody as well as articulation [39,44,45]. Vocal function utilizing voice-voiceless contrasts in speech was found to progressively deteriorate in a group of PWP who had undergone DBS, but this was attributed to disease progression and reduction of medication, not to the DBS therapy [46]. In contrast to these reports in improved or non-affected vocal function, vocal fold closure, accompanied by breathy and strained vocal quality, was reportedly negatively affected by DBS in a large group of PWPs [47].

The dissociation between speech and (other) motor measures is compounded by conflicting subjective reports from persons who have been treated with deep brain stimulation [48,49]. In an extensive study, acoustic measures showing improvement with deep brain stimulation were at odds with perceptual ratings by PWPs and their physicians [50]. In a study of personal impressions using the Voice Handicap Index (VHI) before and after surgery, self-reports describing speech were more variable in PD with than without stimulation [51].

To illustrate these considerations, we recount comments from one of our study participants, during his monologue produced as part of our clinical evaluation:

“Um, somehow my mouth feels like it’s, uh, in the way of my words rather than helping me speak ‘em. And, uh, I feel a little bit of slur, uh, which is, uh, interesting ‘cause it pretty much started this way after I had the DBS hooked up again.”

## Speech Task Effects

Traditionally, the speech disturbance in PD was attributed to “neuromuscular abnormalities in much of the speech musculature, usually related to restriction in the range or speed of movement patterns” [52]. Although variability in movement and speech symptoms was noted [53], it was generally held that “the motor control problems are present regardless of tasks or context” [54]. Dysarthria was characterized by “highly consistent articulatory errors” [54]. Consistency of motor features across speech tasks was emphasized, so that persons with dysarthria show “very little difference in articulatory accuracy between automatic-reactive and volitional purposive speech” [55]. This assumption, that the characteristic signs of dysarthria occur uniformly across speaking conditions, influenced current assessment protocols, which utilize reading or repetition to estimate overall speech intelligibility [56,57].

Despite these perspectives published in the standard literature, acoustic differences as a function of speech task between spontaneous speech and reading were reported [58-60]. A similar role of repetition, contrasting in speech measures with spontaneous speech, was also noted [61-63]. In two individuals with severe dysarthria as a consequence of PD, repetition, reading and sung speech, when compared to spontaneous speech, yielded different outcomes in intelligibility and acoustic measures [64,65]. It appears that speech produced during reading or repetition differs systematically from spontaneous speech. These studies all point to the conclusion that perceptual and acoustic measures derived from repetition or reading [66-68] may not mirror spontaneous speech [69]. As reading and repetition have yielded similar results in comparison to spontaneous speech, the current study focused on compared measures for repetition and spontaneous speech.

## Materials and Methods

### Speech samples

Speech samples were obtained from six right-handed PWPs, all males, diagnosed with non-tremor predominant PD and treated with bilateral deep brain stimulation of the subthalamic nucleus (DBS-STN). Medication and PD rating data are presented in Table 1. Mean age was 58 (range 56-62), mean years of education was 16.1 (range 15-18); mean age of diagnosis was 47.0 (40-51), averaging 11.2 years post PD diagnosis [9-15]. Less demographic uniformity within the group members is seen in one parameter, months since DBS, where the mean number of months was 20.0 and the range was 2-56. Participants were American English speakers with normal hearing by self-report with no other medical or neurological diagnoses and no previous history of speech or language disorders. All were diagnosed with mildly dysarthric, hypokinetic speech including hypophonia, imprecise articulation, dysfluencies, and rate abnormalities, consistent with the diagnosis of PD.

### Procedure

Three sets of speech samples were obtained. First, PD participants provided five minutes of spontaneous speech, from which utterances were taken and randomized for a repetition task. This allowed for closely matched exemplars of spontaneous and repeated speech. Secondly, in a continuous repetition of an utterance, participants produced a challenging 4-word sentence (Pop the top cop) repeatedly for a period of 60 s at two separate sessions. Third, 60 s of spontaneous speech was obtained at two separate times, resulting in 2 min samples. All speech samples were digitally recorded and subjected to analysis. Recordings were obtained with the stimulators turned on and again with the stimulators turned off during separate testing sessions at least one week apart. All recordings were made at least 12 h following the last dose of PD medication.

### Listening test

The listening test provided the first measure in this study: Intelligibility. The listening test, utilizing materials from the first speech sample (5 min of spontaneous speech), was designed so that repeated and spontaneous utterances did not both appear to the same listener. One hundred seventy utterances were randomized and presented in each version. Two significant departures from the previous study were introduced [37]: First, no linguistic support was provided in the transcription exercise; listeners were instructed to transcribe each entire excerpt. Secondly, after adjusting the headphone volume to a comfortable listening level for each listener, the playback was reduced by 7.2 dB to mimic the lower volume of PD speakers [70,71]. Thirty native English speakers (25 females, 5 males) served as listeners. Their mean age was 37.9 ± 17 years and the mean education level was 16.1 ± 2 years. All listeners were born and received their primary and secondary school education in the USA. All research subjects provided informed consent in accordance with the Helsinki declaration of 1975 (and as revised in 1983).

## Measures

### Intelligibility and difficulty

Listeners’ performance was scored as the percentage of correctly transcribed words. Difficulty ratings on a scale from 1-5 were also obtained for each speech sample.

### Acoustic analysis

Measures included the fundamental frequency (F0) mean and coefficient of variation (CoVar; a measure of the variability in the intonation contours) and the voice harmonic-to-noise (HNR) ratio. HNR is a reflection of efficiency of vocal fold vibration as filtered by the vocal tract in the form of periodic and aperiodic signals [72], calculated over voiced portions of the excerpts. Other measures were two indicants of vocal fold vibratory stability, jitter (frequency perturbation) and shimmer (amplitude perturbation) using Praat [73]. These analyses were performed on spontaneous and repeated excerpts, the 60 s continuous phrase repetition task and the 2 min spontaneous speech samples.

### Fluency ratings

Two independent raters identified vowel distortions, consonant substitutions and word and sound omissions derived from the spontaneous and repeated excerpts, from the utterance repetition (Pop-the-top-cop) task and the 2 min of spontaneous speech. Discrepancies between raters were adjudicated by a third rater, all trained in speech science and acoustic phonetics.

## Results

### Spontaneous versus repeated utterances

#### Intelligibility

**Figure 1:**
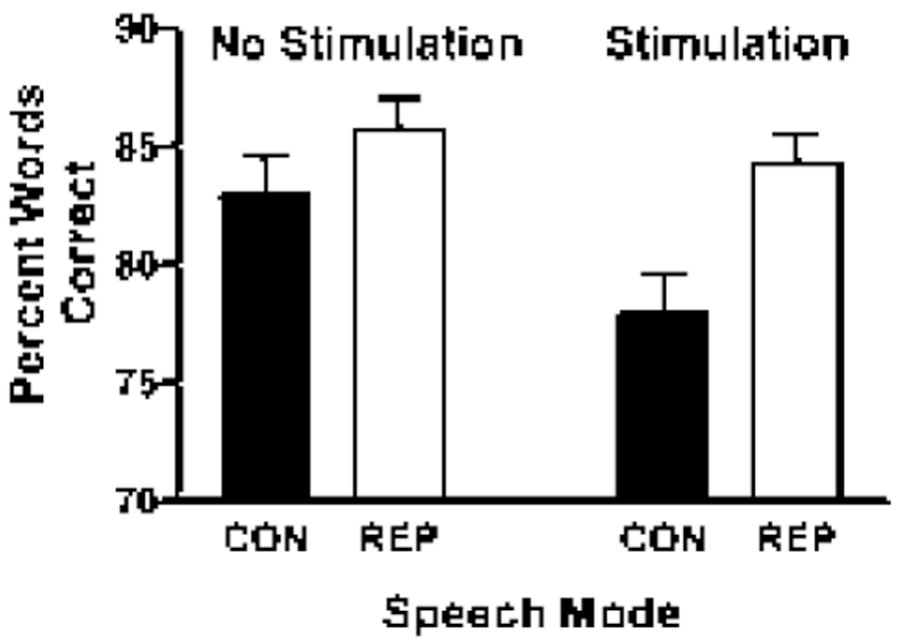
Percentage of words correctly transcribed by listeners for both stimulation states, on and off, in conversation and repetition modes in the first speech sample.

In the listening study, fewer words were correctly transcribed when the basal ganglia were stimulated [*F* (1,29)=9.462; *p*=0.005]. When the two tasks, conversation and repetition, were compared, fewer words were correctly transcribed from spontaneous than repetition [*F* (1,29)=14.06; *p*=0.001]. These two conditions, stimulation state and task, interacted as well [*F* (1,29)=4.543; *p*=0.042]. Postdoc comparisons revealed that more words were correctly transcribed from repetition than from spontaneous speech during the stimulated state [*t* (29)=-2.342; *p*=0.026] and during the off state [*t* (29)=-3.619; *p*=0.001]. In contrast, six percent fewer words were transcribed from spontaneous speech with subthalamic nucleus stimulation on compared to off [*t* (29)=3.098; *p*=0.007], but there was no stimulation effect on words transcribed during repetition (Figure 1). There was a 10% increase in the difficulty ratings for transcriptions with stimulation on compared to off [*F* (1,29)=4.374; *p*=0.045]. Transcriptions from spontaneous speech were rated 11% more difficult than those from repetitions [*F* (1,29)=23.416; *p*<0.001]. These two conditions interacted as well [*F* (1,29)=13.481; p=0.001]. Pairwise, the pattern of difficulty rating differences reflected the pattern for intelligibility. Transcriptions from spontaneous speech were rated more difficult than transcriptions from repetitions in the on [*t* (29)=5.937; *p*<0.001] and off [*t* (29)=2.435; p=0.021] states. Transcriptions from spontaneous speech were also rated as more difficult with stimulation on compared to off [*t* (29)=-3.711; *p*=0.001], but this was not found for repetitions.

#### Acoustic measures

Mean F0 was higher during DBS stimulation [*F* (1,59)=4.361; *p*=0.041]. The same main effect was found for median F0 [*F* (1,59)=5.206; *p*=0.026] and minimum F0 [*F* (1,59)=4.591; *p*=0.036]. Spontaneous speech produced higher minimum [*F* (1,59)=42.577; *p*<0.001] and maximum F0 values [*F* (1,59)=9.811; *p*=0.003].

**Figure 2:**
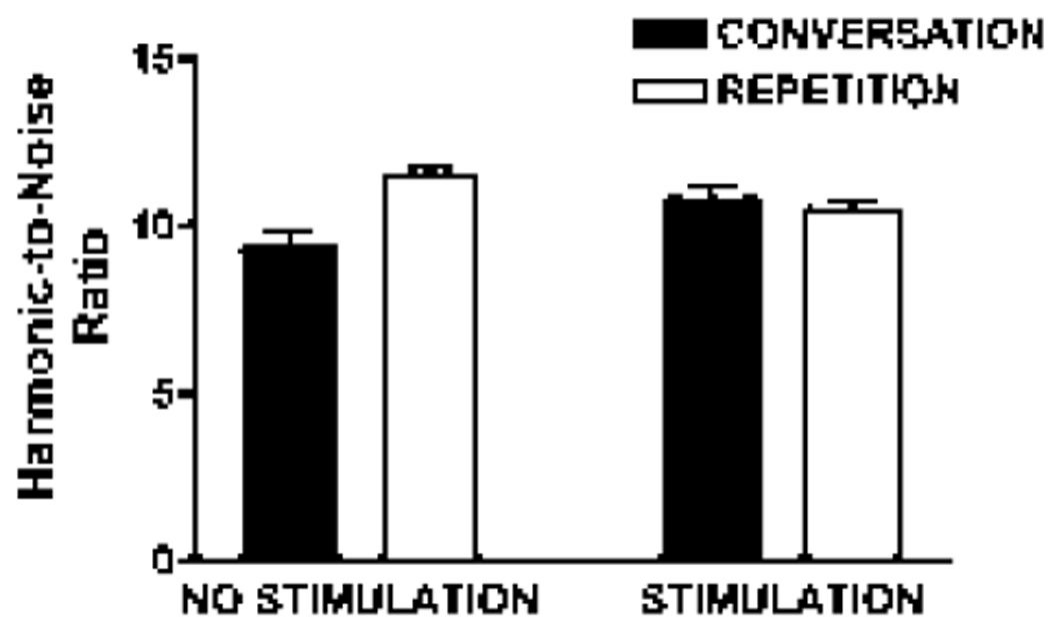
Harmonic to noise ratios in deep brain stimulation on and off for two tasks, conversation and repetition, for both stimulation states, on and off, in conversation and repetition modes in the first speech sample.

Harmonic-to-noise ratio (HNR) was higher on repeated stimuli compared to those taken from spontaneous speech [*F* (1,59)= 9.88; *p*<0.001] and task interacted with stimulation status [*F* (1,59)=19.05; *p*<0.001]. During spontaneous speech, HNR was higher with stimulation on compared to off [*t* (59)=-2.8; *p*=0.007]. In contrast, HNR during repetition was higher off compared to on [*t* (59)=3.11; *p*=0.003]. With stimulation off, HNR was higher during repetition compared to spontaneous speech [*t* (59)=-5.86; *p*<0.001]. When deep brain stimulation was on, this difference was eliminated with spontaneous speech HNR improving to the level of repetition. These measures reflect the consistent findings of a sturdier voice, first, during repetition and secondly, with stimulation (Figure 2).

**Figure 3:**
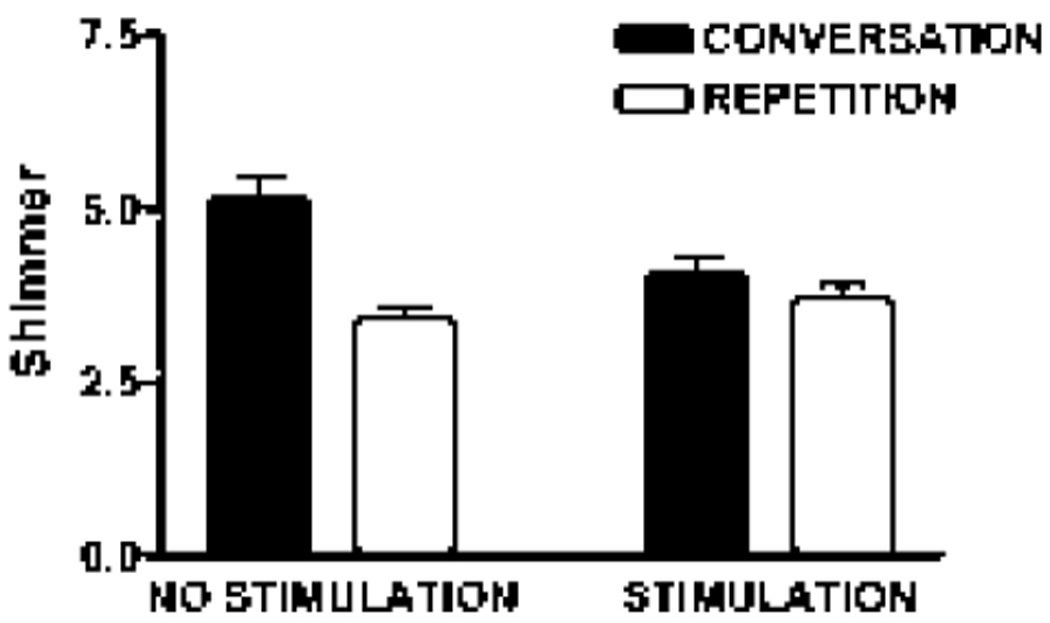
Shimmer measures in deep brain stimulation on and off for two tasks, conversation and repetition, in the first speech sample.

Shimmer was higher during spontaneous speech compared to repetition [*F* (1,59)=19.8; *p*<0.001], and task interacted with stimulation status [*F* (1,59)=8.52; *p*=0.005]. During conversation, shimmer was higher with off compared to on [*t* (59)=2.67; *p*=0.01]. Shimmer did not differ as a function of stimulation status during repetition. In the off state, shimmer was higher during spontaneous speech compared to repetition [*t* (59)=4.67; *p*<0.001]. These measures also suggest that the spontaneous speech mode and the absence of stimulation both contribute to a less stable vocal signal. Neither task nor stimulation state had significant effects on jitter measurements (Figure 3).

#### Fluency measures

The text excerpts utilized by the listeners for the intelligibility measure were examined for dysfluency. The numbers derived from these short speech samples were too sparse for statistical analysis. The overall raw tally of dysfluencies was, for stimulation on, repetition 6 and spontaneous speech 28; for off, repetition 8, spontaneous speech 20, mirroring other findings for increased dysfluency in spontaneous as compared with repeated samples, as well as increased dysfluency during stimulation.

## Continuous Sentence Repetition *(Pop the Top Cop)*

### Acoustic measures

**Figure 4:**
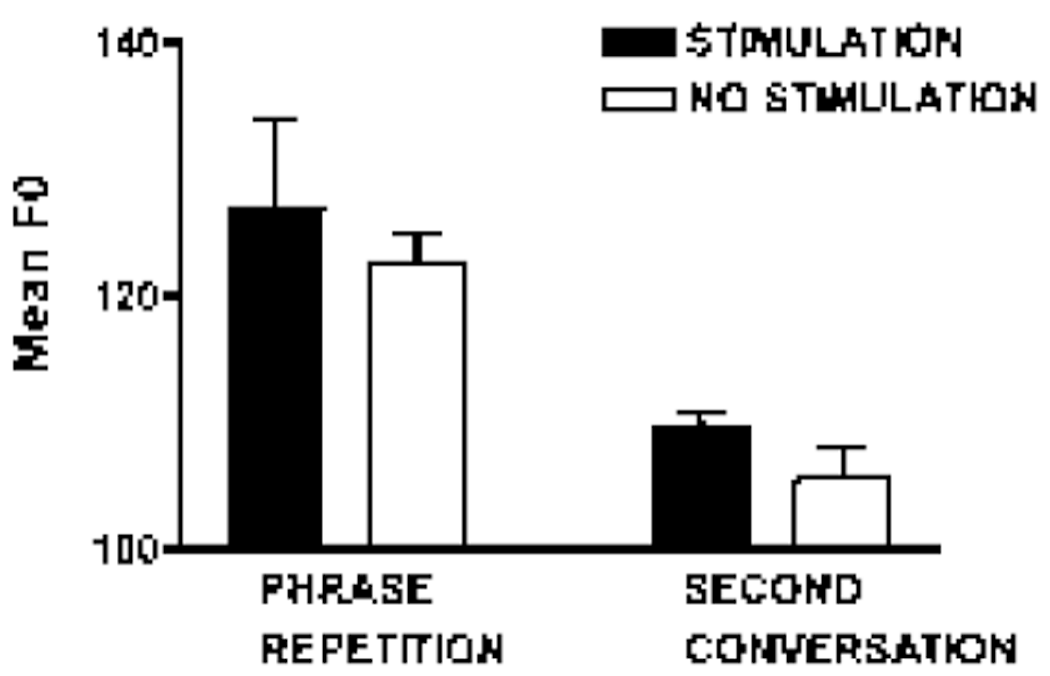
Mean F0 in two stimulation states, on and off, in continuous phrase repetition and the second conversational sample.

**Figure 5:**
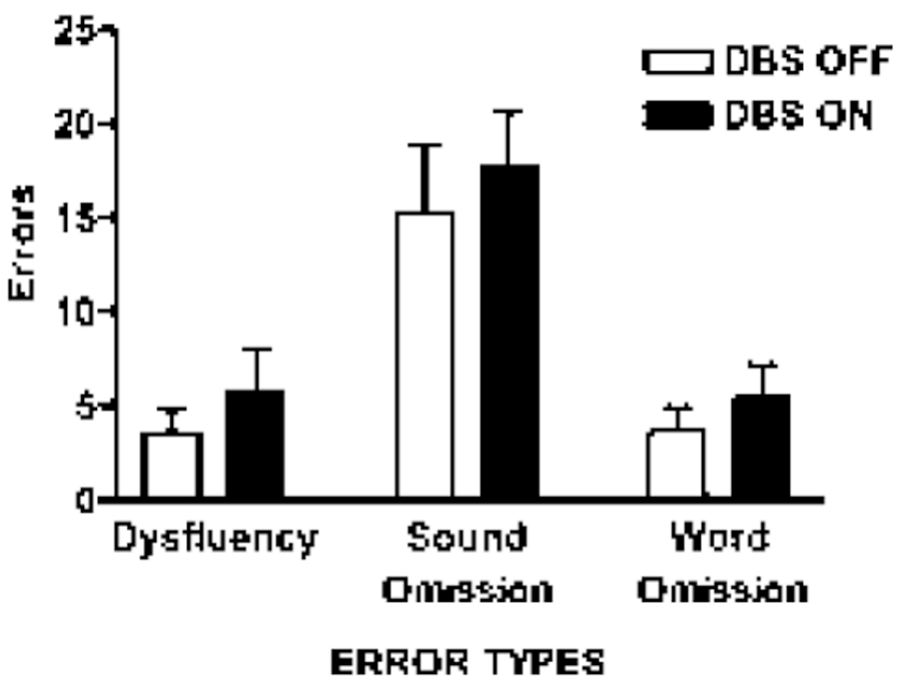
The number of motor speech errors in three categories during the continuous repetition of the sentence /pop the top cop/. The categories were dysfluency (e.g. repetition of a phone or word), sound omission and word omission.

**Figure 6:**
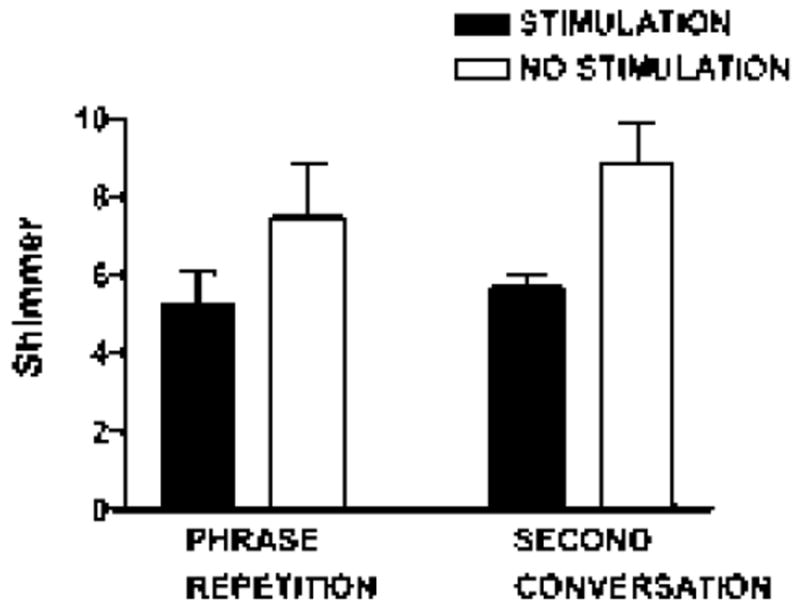
Shimmer values during two stimulation states, on and off, for continuous phrase repetition task and the second conversational sample.

On the continuous sentence repetition task (/*pop the top cop*/), mean F0 increased 3.6% [*t* (9)=-3.31; *p*=0.009], and HNR increased 20.7% with stimulation [*t* (9)=-3.06; *p*=0.014]. Shimmer (11 point amplitude perturbation quotient) was reduced by 30% with stimulation [*t* (8)=2.98; *p*=0.018] (Figures 4-6).

### Fluency measures

For the continuous utterance repetition task (*pop the top cop*), the numbers of speech errors in three categories dysfluencies, sound omission and word omission were assessed (Figure 5). Taking into account the duration of PD as a covariate, there was a significant effect of stimulation [*F* (1,12)=6.06; *p*=0.03], with 28.9% more errors in the on condition across categories (on: 9.64; off: 7.48). Pairwise, there was a significant interaction between stimulation state and error type, with the greater effect observed for dysfluencies [*F* (1,12)=5.85; *p*=0.032]. Further, there were a greater number of vowel distortions with stimulation off compared to the on condition [*F* (1,54)=5.437; *p*=0.023], implying insufficient articulatory posturing with stimulation on (Figure 5).

### Min spontaneous speech sample

#### Acoustic measures

On the second spontaneous speech sample, mean F0 increased 3.9% with stimulation on, comparable to the increase observed in the repetition task [*t* (5)=-2.59; *p*=0.049]. HNR did not differ, but shimmer was reduced by 36% with stimulation [*t* (5)=2.73; *p*=0.041] (Figures 4 and 6).

#### Fluency measures

There were significantly more dysfluencies produced with stimulation on compared with off measured across two minutes of spontaneous speech [*F* (1,40)=6.586; *p*=0.014].

## Discussion

Both stimulation state and speech task influenced intelligibility, difficulty ratings, acoustic measures, and fluency ratings. The most notable declines in intelligibility and fluency were observed for spontaneous speech during stimulation.

The effects of stimulation and task on listeners’ difficulty ratings were similar to those for intelligibility. Difficulty ratings were higher for spontaneous speech than for repeated speech in both stimulation states.

The dysarthria of PD speakers typically includes higher shimmer and jitter, lower HNR, lower fundamental frequency, and intensity variability [52,74,75]. An improvement in vocal fold functionality, including F0, jitter and tremor, has been described following levodopa therapy [76], often viewed as analogous, in its effects, to stimulation treatment. Results from the present and comparable studies suggest that stimulation treatment offers an amelioration of these vocal disabilities without necessarily improving intelligibility.

Improved vocal quality during stimulation was also demonstrated by increased harmonic-to-noise ratio, a measure of periodicity in voice quality and quantitative indicator of degree of hoarseness [77], for spontaneous speech. Similar effects have been reported previously. In a case study of a person with PD and severe dysarthria, a noisier, more aperiodic signal was seen in spontaneous speech than in reading, repeated speech or singing [64].

Voice measures improved in the same manner for the continuously repeated utterance, *Pop the top cop*, and for the second spontaneous speech sample. F0 was higher in the stimulated state, suggesting stiffening effects on vocal fold function. Shimmer was reduced, implying less variability in vibratory processes. Finally, harmonic-to-noise ratio was higher in the utterance repetition task with stimulation, implying greater periodicity in the signal arising from vocal fold vibration. This consistent array of improved voice measures in association with deep brain stimulation strongly suggests a subcortical influence on laryngeal deportment, possibly with the aid of more consistent respiratory control.

Despite improved measures for voice, intelligibility was overall mildly depressed following stimulation, especially for spontaneous speech. This dichotomy was also found in another study, which reported better vowel quality in the stimulated state without greater speech intelligibility [78]. It appears that stimulation improves vocal parameters, but it does so in the context of poorer intelligibility, for which compromised articulatory parameters provide a likely explanation.

In an earlier study, PD speakers were found to be more dysfluent with stimulation [79] and two cases of dysfluency following deep brain stimulation have been reported [80]. Stimulated speech contained more intraphrase pauses than non-stimulated speech [81]. In the current study, vowel distortions, attributable to articulatory insufficiency, and more lexical and phonological dyfluencies were documented under stimulation than off stimulation in all the samples examined. Previously, clinical ratings were higher on vocal parameters (better voice quality) and lower on dysfluencies (greater fluency) for repetition than spontaneous speech [37]. A concomitant discrepancy between positive effects on voice and negative effects on fluency has been reported elsewhere [3,24]. An overall profile suggests that voice characteristics, weak in untreated PD, are stronger during repetition and are enhanced with subcortical stimulation, while fluency is reduced in spontaneous speech. This combination of strong vocal production driving diminished articulatory control may account for the impression of difficulty with talking reported by persons with PD as well as for the lowered intelligibility in spontaneous speech and subcortically stimulated speech.

The motor planning framework may provide one of the keys to understanding intelligibility effects with respect to acoustic changes in speech following stimulation. Evidence comes from a study on the effect of stimulation on vowel space [82]. Individuals with PD on and off stimulation were compared with healthy control speakers producing sustained vowels. Vowel space was determined for the initial 250 ms as well as the midpoints of each vowel. There were no significant differences in the midpoint vowel space measurements between on and off states and control values. However, in the control and off conditions, the initial vowel spaces were significantly larger than the midpoint vowel spaces. In contrast, on stimulation, the initial vowel space was significantly smaller than in the off and control conditions. It appeared that deep brain stimulation may alter vocal tract posturing at the initiation of production. These results are consistent with the findings in the current study, in which articulatory imprecision is enhanced by deep brain stimulation.

The present results also demonstrate that in addition to stimulation status, task affects speech and that these two factors interact. Several studies have revealed significant differences in speech measures taken from spontaneous speech in comparison with reading or repetition in PD [65,83,84] in dysarthric [85] and cerebellar speech [61,63]. In a presentation of severe stuttering in a person diagnosed with Parkinsonian syndrome, repetitions and prolongations were 200% greater in spontaneous speech than in reading, repetition or singing [65]. Using four tasks in a previous study, automatic speech, elicited speech, spontaneous speech and reading aloud, significant differences in habitual loudness or pitch were found when performance measures were compared [86,87]. In another study, vowels were extracted from sustained phonation, sentence repetition, reading, and monologue and submitted to detailed acoustic analysis. Spontaneous speech was the most sensitive in differentiating between controls and PD patients, suggesting that articulatory difficulties are more likely to emerge in spontaneous speech than in structured tasks [88-90]. Acknowledging the significance of task effects in clinical evaluations, an algorithm to systematically relating measures from spontaneous speech with those from repetition, in terms of scaled estimates of intelligibility, was developed [69].

It has been proposed that cognitive-linguistic load is accountable for lower intelligibility and weaker articulatory performance in spontaneous speech, but this view has not been verified by empirical study. Attempts to document a distinction in reading versus spontaneous speech measures based on a controlled cognitive-linguistic contrast have not been successful; task-related differences using reading and narrative speech did not differ between low-cognitive and high-cognitive groups with multiple sclerosis, using pausing and speech rate as measures [91,92]. It is arguable that reading and repetition also place cognitive demands on the speaker, for example, in requiring the pronunciation of relatively unfamiliar elements and structures and demands on short-term memory. In addition, it is difficult to see how lower cognitive demands (alleged by this theory to be inherent in reading and repetition) can be associated with improved voice measures, as has been consistently documented.

Instead, it is plausible that reading and repetition both provide an external model of the desired speech output while spontaneous speech requires the generation of an internal model of the motoric gesture. An external model reduces the burden on the motor speech neurological apparatus, which can proceed with less demand for initiation, sequential planning, and monitoring, thereby facilitating more efficient motor execution [88,93]. The observation that PD was associated with deficient recited speech (e.g. Humpty Dumpty), when compared to healthy speakers, further supports the hypothesis that highly routinized or procedural speech sequences with well-established internal models are impaired by basal ganglia dysfunction [94-98]. This perspective places task effects more solidly at the level of motor organization, initiation, planning, monitoring, and execution, leaving aside concerns about linguistic or cognitive characteristics. Support for this view can profitably be considered in the light of findings from gait and arm movement studies [99-101] whereby providing a model significantly improves execution of the movement [102-104]. Motor deficits in Parkinson’s disease are more severe in internally than in externally guided motor tasks [105,106] implying that, for speech, a deficient subcortical system can be expected to perform more poorly for spontaneous speech, when a newly generated internal model is required, than in repetition or reading, where an external model is provided.

## Conclusion

This study examined Parkinsonian speech under stimulation on and off conditions to investigate subcortical influences on details of voice and articulation. Stimulation, allowing for enhanced subcortical function in basal ganglia disease, affected both of these components of motor speech, but in an orthogonal manner. Improvement in voice quality was accompanied by a reduction in fluency. Overall, intelligibility was reduced in the stimulated state. A stronger laryngeal component may be competing with a less competent articulatory system, leading to a compromised motor speech product and an impression of greater effort, in the PD speakers themselves, in the activity of speaking, as reported by one of our participants in the beginning of this report. From a speech motor control perspective, improvements in voice characteristics may have a disproportionate effect on speech, altering the parameters of the intended speech output most disruptively in spontaneous speech, when no external model is present. Analogously to gait and arm reach, the repetition mode provides an external model, aiding motor output efficiency in subcortical processing for speech.

## Figures and Tables

**Table 1:** Overview of demographic data for study participants.

PD (years)	DBS (years)	Levodopa	H&Y Off	H&Y On	UPDRS-III Off	UPDRS-III On
10	9	600	2.5	2.5	27.5	19.0
15	2	600	2.5	2.0	26.0	23.0
11	4	600	2.0	2.0	14.0	1.0
9	4	300	2.5	2.0	23.5	21.5
11	56	600	4.0	3.0	52.5	31.0
11	37	400	2.0	1.0	11.5	4.0

ED: Education; PD: Parkinson’s Disease; DBS: Deep Brain Stimulation; H&Y: Hoehn and Yahr rating scale for PD; UPDRS: Unified Parkinson’s Disease Rating Scale

The stimulator frequency was 185 Hz and the pulse width was 60 μs in all cases
